# Professional Development for Teachers in the Digital Age: A Comparative Analysis of Online Training Programs and Policy Implementation

**DOI:** 10.3390/bs15081076

**Published:** 2025-08-07

**Authors:** Yuanhai Gu, Jun He, Wenjuan Huang, Bo Sun

**Affiliations:** 1 School of Future Education, Beijing Normal University, Zhuhai 519087, China; g.y.hardy@mail.bnu.edu.cn (Y.G.); junhe1133@163.com (J.H.); 2 Faculty of Artificial Intelligence in Education, Central China Normal University, Wuhan 430079, China; hjuanjuan@mails.ccnu.edu.cn

**Keywords:** teacher professional development, online training, digital education, policy implementation, fair education, comparative analysis

## Abstract

In the digital age, online teacher professional development (TPD) has become a key strategy for enhancing instructional quality and ensuring equitable access to continuous learning. This research compares and analyzes Chinese online teacher professional development (TPD) with the United States over a period of ten years, from 2014 to 2024. This study uses a mixed-methods approach based on policy documents, structured surveys, and interviews to investigate how governance regimes influence TPD outcomes for fair education. Both countries experienced a massive expansion of web-based TPD access and engagement, with participation rates over 75% and effectiveness scores over 4.3 by 2024. China focused on fast scaling by way of centralized mandates and investments in infrastructure, while the United States emphasized gradual expansion through decentralized, locally appropriate models. Most indicators had converged by the end of the period, even with these different approaches. Yet, qualitative evidence reveals persisting gaps in functional access and contextual appropriateness, especially in rural settings. Equality frameworks with attention to teacher agency, policy implementation, and digital usability must supplant weak access metrics. A hybrid paradigm presents itself as an attractive means toward building equitable and productive digital TPD environments through the symbiotic integration of China’s successful scalability and the United States’ professional autonomy.

## 1. Introduction

The swift development of technology and evolving needs of learners in the twenty-first century are bringing dramatic shifts in the education sector in the present digital world. The central force behind it is the revolution in teacher professional development (TPD), which has moved from traditional, face-to-face workshops and seminars to more vibrant, flexible, and accessible online systems. Professional development online is now crucial in providing teachers with the digital savvy and skills they must have to keep up with evolving curriculum, emerging teaching resources, and the diversified needs of learners. This development has been hastened by the COVID-19 pandemic, which has forced global educational systems to rethink inclusive, agile, and disruption-ready professional learning models (Brown et al., 2021).

Due to this demand, governments and educational institutions, respectively, have put in place digital training programs and upgraded national policy frameworks, which has made online learning an effective strategy for professional capacity-building. The structure, length, material focus, delivery (e.g., synchronous, asynchronous, or blended), and institutional support provisions differ widely amongst these programs. Despite the perceived benefits of online TPD, which includes enhanced accessibility, cost-effectiveness, and scalability, there are concerns about its effectiveness, equity, and compliance with educational standards (Djatmiko et al., 2025). Teachers often find disparities in access, digital infrastructure, technological readiness, and institutional support when using these programs, particularly in low-resource environments.

The notion of equitable education has emerged as an essential framework for assessing online TPD. Equitable education transcends mere equal treatment; it highlights the need for fair opportunities, acknowledging that various teachers may need distinct forms of support to thrive. (Anderson, 2007). From this perspective, professional development programs must be available but also accessible, inclusive, and responsive to contextual needs. However, digital divides defined by variations in internet connectivity, access to devices, and digital literacy can exacerbate existing inequities and undermine the goals of educational justice (Memon & Memon, 2025). These challenges are exacerbated by gaps in policy design and implementation, especially in nations where national strategies fail to align with local practices and where professional development lacks quality assurance and regular evaluation mechanisms.

Although the importance of online TPD is increasing, the current literature focuses mainly on individual program evaluations, satisfaction surveys, or technological aspects that are often restricted to specific countries or institutions. There remains a significant gap in comparative research that examines how national or regional policy frameworks affect the design, execution, and equitable outcomes of digital professional development programs. Comparative insights are crucial in order to identify best practices, examine systemic barriers, and develop evidence-informed recommendations aimed at improving the quality and equity of TPD worldwide (Asterhan & Lefstein, 2024).

This study will compare online teacher professional development programs and their surrounding contexts or policies as a means to further bridge the gap and examine how the latter can further the cause of equality in education. The aim of this research is to explore how online TPD design, delivery, and accessibility vary across countries or institutions and how the digitalization of teacher learning is configured across systems. Simultaneously, the study assesses whether legislation impacts programs, and if current legislation makes it feasible to access equitable, inclusive, and worthwhile professional development.

There are three interrelated goals for this study. This paper first examines formats, platforms, target audiences, and content to explore how online teacher professional development (TPD) programs differ in their structure, delivery methods, and accessibility across different institutions and national contexts. Second, with an emphasis on finance, monitoring, implementation tactics, and institutional coherence, it evaluates how present education policies either facilitate or impede fair access to these programs. Thirdly, it investigates how instructors view the efficacy and equity of online TPD, with a focus on the infrastructures and policy environments that shape these initiatives. This study compares the impact of digital policy environments and online training programs on teacher professional development (TPD) outcomes in China and the United States. It explores program design, governance, and teachers’ experiences to understand how digital innovation and policy implementation relate to equitable education within teacher learning. The results are intended to serve as a guide for teachers, legislators, and development providers regarding the systemic requirements for developing inclusive, superior, and long-lasting online training models for teachers in the digital age.

## 2. Literature Review

### 2.1. The Evolution of Teacher Professional Development in the Digital Age

It is commonly acknowledged that enhancing student learning outcomes and the quality of instruction requires teacher professional development, or TPD. TPD has traditionally been provided through top-down, in-service training, workshops, conferences, and institutional seminars, which have resulted in low levels of involvement and relevance to local contexts (Hennessy et al., 2022). Despite their critical role in skill transfer, these models often fail to encourage teachers to engage in continuous, introspective, and transformative learning. 

As a result of the rise of digital technology, TPD has quite a few chances to be reconsidered. With the advent of online learning platforms, mobile applications, online communities, and open educational resources, the alternatives and approaches available to teachers in order to undergo professional development have increased (Klein & Ware, 2003). The change required is to go from a norm model to a more immediate, adaptable, and personalized model of TPD. Through asynchronous learning, micro-credentialing elements in webinars and interactive forums, teachers can now learn at their own pace and apply knowledge in real-time (Aithal et al., 2024). The COVID-19 pandemic uncovered structural deficits in the architecture of professional learning. It also highlighted the importance of digital competence. So, this transition accelerated significantly. As schools closed to halt teaching, educational systems seamlessly switched to using online professional development (OPD) platforms around the world. In this situation, OPD actuated not only as a stopgap measure but also as a test site for environment-friendly and scalable teacher learning strategies in the digital age (Bragg et al., 2021).

### 2.2. Effectiveness and Design of Online Teacher Training Programs

Beyond technology adoption, other factors (e.g., successful instructional design, engagement tactics, and contextual integration) also determine the success of online TPD. Research indicates that formative feedback, such as working with peers, mentoring, active learning, and the opportunity to practice in the classroom, is crucial to effective OPD programs, which have a significant impact on teachers’ practice (Bragg et al., 2021). The principles of adult learning theory highlight the importance of being self-directed, relevant, realistic, and experiential. The most successful online TPD encourages interaction, continuous engagement, and reflection practice, based on a thorough meta-aggregative review published in ‘Educational Technology Research and Development’ by Philipsen et al. (2019). Moreover, fostering an environment of meaningful professional discourse in virtual spaces hinges on a strong community of inquiry considered to include cognitive, social, and teaching presences (Shea & Bidjerano, 2009).

Commonly referenced resources include discussion boards, live sessions, group projects, digital portfolios, and other useful tools that can enhance authenticity and involvement (Dailey-Hebert, 2018). But there are still difficulties. Teachers who use OPD platforms may present constraining factors, such as time allocation, deficient digital competence, technical glitches, and motivational fatigue, particularly within poor-resourced settings. Moreover, the use of purchased concepts may be problematic when TPD materials focusing on such concepts do not match the local teaching context and school curriculum. Tackling these problems means relevant choices related to design principles and institutional support structures that will enhance successful engagement and knowledge use.

### 2.3. Policy Frameworks Supporting Digital TPD

The effectiveness of online educator development depends not only on the design of these courses, but also on the policy landscapes in which they are embedded. National policies on education, digital initiatives, and teacher competency frameworks shape the design, finance, and implementation of TPD systems. In different countries, many governments have launched strategic plans that contain requirements for digital training, standards for professional learning, and a centralized learning platform. As an illustration, the Digital Education Action Plan (2021–2027) published by the Commission aims to develop effective, digitally competent teaching in the EU member states (Outeda, 2024).

In systems such as Finland and Singapore, which have exceptionally high-quality education, the policy integrates OPD within their ecosystem, such as teacher agency, professional learning communities, and continual coaching (Goodwin et al., 2017). These policies are characterized by coherence, long-term vision, and strong institutional alignment. In contrast, many developing countries face implementation bottlenecks, where weak infrastructure, funding gaps, and insufficient monitoring mechanisms undermine policy ideals. Implementation fidelity, defined as the degree to which policies are executed as intended, varies widely. Research in computers and education, the Internet, and higher education highlights that without inter-organizational coordination and school-level leadership, even well-crafted policies may fail to translate into meaningful learning experiences (Goodyear & Carvalho, 2014). Therefore, beyond policy formulation, capacity building, stakeholder engagement, and policy adaptation at the local level are essential to drive effective and equitable online TPD.

### 2.4. The Role of Fair Education in Teacher Professional Development

The concept of fair education, grounded in social justice theory, entails the removal of structural barriers that impede equal access to learning opportunities. Within the realm of teacher development, fairness implies that all teachers, regardless of geographic location, socio-economic background, gender, or institutional type, should have access to high-quality professional learning that responds to their needs. This requires inclusive program design, policies centered on equity, and focused resource allocation.

Research reveals that teachers still face access and preparedness gaps in using digital tools. It is not uncommon for teachers working in schools which are underfunded, in rural areas, or where the dominant language spoken is non-dominant, not to have the resources, internet access, or assistance to benefit from OPD (Kachikwu, 2023). Moreover, women teachers may experience comparatively larger fallout from issues of gender and care relationships that limit their visibility, time, and flexibility to navigate digital platforms (Gandolfi et al., 2021). The academic literature broadly supports the use of a universal design for learning (UDL) approach to digital teacher professional development (TPD) to address the needs of diverse learners with engagement, representation, and expression strategies. International frameworks, such as Education 2030 and Sustainable Development Goal 4 (SDG 4), emphasize the importance of scalable, equitably distributed, and culturally relevant professional development systems.

Nonetheless, these pressing issues have yet to find specific redress in national policies or OPD actions. The differences in learning outcomes are often overlooked due to the evaluation of TPD programs which focus more on reach and efficiency rather than inclusion and depth. Policymakers and education researchers must include fairness as a fundamental criterion alongside quality and effectiveness.

### 2.5. Gaps in the Literature and the Need for Comparative Research

Despite increased scholarly interest in the topic of online teacher development (OPD), the body of the existing literature remains fragmented along methodological, disciplinary, and geographic lines. Many studies ignore regulatory frameworks or systemic constraints in favor of concentrating only on the technical components of OPD programs or teachers’ views. Furthermore, the majority of assessments are conducted in settings that are exclusive to a single nation or institution, which limits the possibility of cross-contextual learning and policy adaptation.

To find trends, differences, and contextual factors that influence success in various educational systems, comparative study is crucial. The importance of comparative frameworks in identifying structural facilitators and barriers that affect the results of digital education is highlighted by journals like *Compare: A Journal of Comparative and International Education* and *International Journal of Educational Development*. Few studies take an integrated strategy that connects educational fairness, policy implementation, and online program design. This gap inhibits our understanding of how different systems successfully or unsuccessfully align the ideals of equitable education and their infrastructure for professional learning. To fill this gap, empirical research that looks at what works and for whom, in what context and under what conditions, is needed.

Switching to online teacher professional development has presented new opportunities and challenges for educators, institutions, and politicians. Digital formats can stimulate innovation and democratize access; their effects, however, range quite widely due to variation in institutional support, policy consistency, and design quality. It is also crucial to assess teacher professional development (TPD) on ηνα HηA reach, effect, inclusivity, and responsiveness to different kinds of teachers because all education is fairly given. This study aims at filling an important knowledge gap by assessing the interaction between online TPD programs and their policy frameworks to shed light on how they either support or hinder equitable professional development in a digital age.

## 3. Methodology

### 3.1. Research Design

This research employs a comparative qualitative case study method to investigate the evolution, implementation, and perspectives of online teacher professional development (TPD) programs in China and the United States. It adopts the design approach based on Godsk and Nielsen (2024) and Thurm et al. (2024), emphasizing in-depth, multi-source data collection to understand contextual differences between national TPD systems. Countries were selected based on their innovative practices for technological integration in education (Bhutoria, 2022). Their diverse governance systems also make them important players in global education. The goal of this comparison is to illustrate the impact that variations in policy regime, digital infrastructure, and culture have on the effectiveness and equity of TPD online.

The period spanning from 2014 to 2024 is analyzed to study the long-term trends, changes in policy objectives, implementation strategies, and instructors’ experiences with online TPD. Combining data from national policy documents, structured surveys, and semi-structured interviews allowed for methodological triangulation, which enhanced the validity and dependability of the findings. In alignment with the research objectives, this study is guided by the following exploratory hypotheses:

**H1.**  *Teacher participation in online TPD has increased in both countries over time, with higher rates in the U.S.*

**H2.**  *Better digital access leads to greater participation and perceived fairness in online TPD.*

**H3.**  *Teachers who are more aware of TPD policies perceive them as more effective and equitable.*

### 3.2. Case Selection and Sampling Strategy

China and the USA make for an interesting juxtaposition for this research as they are both large contributors to educational innovation, they have contrasting types of political systems, and they have different regional diversities. The differences thus identified were instrumental in constructing a compelling framework for researching the structure and experience of online teacher professional development (TPD). Both countries adopted stratified sampling techniques for participant choice. A stratified sampling strategy based on surveys and interviews was adopted, stratification was based on key characteristics including teaching subject, school level, years of experience, geographic region, and urban–rural status, following established mixed-methods sampling practices (Creswell & Clark, 2017) Although the stratified sampling design helped lower selection bias, all survey data were self-reported and could be affected by social desirability bias. Participation was voluntary, so responses might underrepresent teachers who are less engaged or lack digital access. No control variables were used; future research should consider employing propensity-matched designs or longitudinal tracking to better explore causality analysis.

This enhanced sampling strategy ensures depth, saturation, and representativeness across multiple levels of the TPD ecosystem.

### 3.3. Data Collection Methods

A mixed-methods approach to data collection was employed to conduct a comprehensive examination of the implementation and experiences of online teacher professional development (TPD) in the United States and China. Structured surveys were implemented to collect quantitative data from a stratified sample of 200 instructors, with 100 instructors from each country. The sample size and diversity were further informed by empirical benchmarks for variability and thematic representation in educational research (Guest et al., 2006). The survey instrument collected data regarding the frequency of participation, accessibility of digital infrastructure, perceived efficacy of TPD programs, policy awareness, and equity of access. To corroborate the survey results, semi-structured interviews were conducted with 50 teachers (25 from each country), 8 education policymakers (4 from each country), and 6 TPD program developers (3 from each country). Deeper insights regarding program design, usability, policy implementation, and professional satisfaction were obtained through these interviews. Furthermore, policy documents, national strategic reports, and institutional guidelines concerning digital TPD were thematically analyzed and reviewed from 2014 to 2024. We acquired a comprehensive comprehension of the creation, communication, and experience of TPD policies in a variety of national contexts by combining survey data, interviews, and document analysis. This comprehensive approach improved the study’s validity and guaranteed that the results were consistent with the systemic objectives and the experiences of practitioners.

To improve transparency and ensure consistency between data collection tools and the study’s analytical framework, the survey and interview questions were organized around five key research constructs. These are participation, access, policy awareness, perceived effectiveness, and equity. Table 1 provides a summary of representative items from both the structured survey and the semi-structured interviews for each construct.

### 3.4. Data Analysis Procedures

The data analysis employed a mixed quantitative and qualitative methodology to identify contextual meanings and statistical patterns within the two national systems. Quantitative survey responses were subsequently subjected to inferential tests, such as independent samples t-tests, and descriptive statistics, such as means and standard deviations, after being cleaned and verified. The objective of this investigation was to compare the participation rates, policy awareness, perceived efficacy, and equality perceptions between the United States and China during specific time periods from 2014 to 2024. In order to facilitate comparison interpretations, cross-tabulations and time-series plots were implemented to illustrate trends. In accordance with the conceptual framework of the study, qualitative data from policy documents and interviews were transcribed and subjected to thematic analysis using both deductive framing and inductive coding. The NVivo 14 software was used to code and control the interview data, which revealed consistent trends and differences in teacher experiences, policy communication, and implementation fidelity. Document analysis procedures were employed to analyze policy texts, which monitored changes in stakeholder roles, strategic priorities, and rhetoric over the past decade. The combination of quantitative and qualitative data improved the interpretation stage of the comprehension of both convergent and context-specific dynamics in online TPD reform practice. Although this analysis used descriptive statistics and t-tests to compare groups, future research could incorporate multilevel regression models to account for nested data structures, such as teachers within schools and schools within districts. This approach would also allow examination of how demographic, geographic, and policy factors interact, offering a more comprehensive understanding of the causal pathways influencing equity and participation.

### 3.5. Instrument Validation

The survey instrument was pilot tested with 15 teachers (not part of the main sample) to confirm clarity and comprehensive coverage of the constructs. Items were organized into scales measuring Cronbach’s alpha values for participation (α = 0.84), perceived effectiveness (α = 0.81), policy awareness (α = 0.79), and perceived equity (α = 0.86), all of which demonstrated acceptable internal reliability. The survey items were adapted from previously validated surveys on professional development and digital equity, ensuring their construct validity across all contexts.

## 4. Results

This section provides a comprehensive comparison of the trends in online teacher professional development (TPD) in the United States and China from 2014 to 2024. The analysis adheres to the equity-driven paradigm of the study and employs descriptive and inferential statistics. Three advanced tables and figures are contextually referenced in this section to improve the rigor and clarity of the analysis.

### 4.1. Trends in TPD Participation Rates and Perceived Effectiveness

The increasing participation rates and the increasing confidence of teachers in the efficacy of online programs are both indicative of the growth of teacher professional development (TPD) in the digital age. Based on the well-structured dataset generated as part of the methodology, this section offers a comparative analysis of perceived efficacy and involvement in the United States and China from 2014 to 2024. By 2024, 85% of teachers in the United States participated in online teacher professional development (TPD), a significant increase from the 45% who did so in 2014. District-level innovations that have enabled digital professional learning options, sustained governmental support, and infrastructure investments are the reasons for this heightened tendency. In contrast, China’s participation rate was 30% in 2014, but it increased significantly, ultimately reaching 75% by 2024. The faster development curve of China is associated with large-scale centralized changes that were implemented after 2018, such as the Smart Education of China project, which rapidly expanded national platforms for teacher training.

Teacher-reported effectiveness of online TPD programs followed similar patterns. In the United States, average perceived effectiveness scores (on a 5-point Likert scale) increased from 3.20 in 2014 to 4.50 in 2024. This reflects the maturation of program design, with greater focus on modular, needs-based, and pedagogically aligned content. In China, effectiveness ratings improved from 2.80 to 4.30 over the same period, with a substantial rise occurring in the latter half of the decade, parallel to infrastructural upgrades and more personalized course offerings. To determine whether the observed differences between the two countries were statistically significant at each time point, independent samples t-tests were conducted using the fixed dataset values. Results are displayed in Table 2. In all selected years (2014–2024), the differences in both participation and effectiveness rates between the U.S. and China were statistically significant, with *p*-values consistently < 0.0001 for participation and mostly < 0.01 for effectiveness. This indicates that while both countries improved over time, the United States consistently maintained higher averages, though China demonstrated notable catch-up in the latter years.

**Figure 1:**
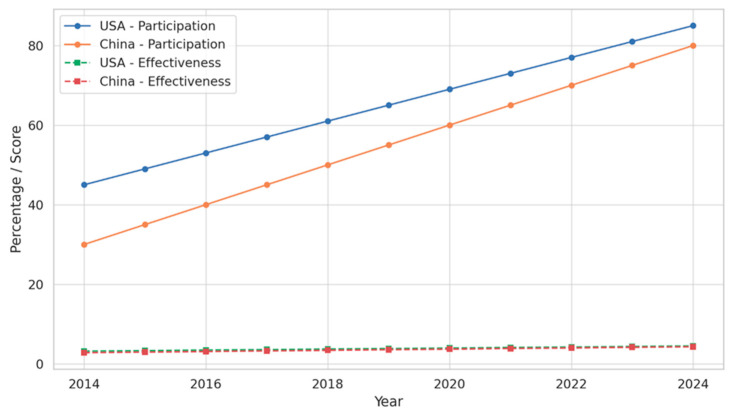
TPD Participation and Effectiveness Trends (2014–2024).

These patterns are illustrated in Figure 1, which shows parallel upward trends for both participation and effectiveness, with China’s steeper curve reflecting a more recent but rapid transformation. The narrowing gap between the two countries by 2024 suggests convergence in TPD outcomes, albeit from distinct starting points and systemic strategies.

### 4.2. Digital Access and Infrastructure

Digital access, specifically, reliable internet connectivity and access to suitable computing devices, is a foundational enabler of online teacher professional development (TPD). Even the most well-designed training programs can exacerbate educational disparities without equitable and sufficient infrastructure. This section presents a comparative analysis of internet and device access among teachers in the United States and China from 2014 to 2024, drawing on the consistent dataset defined in the methodology. In 2014, approximately 85% of teachers in the U.S. had access to a reliable internet suitable for participating in online TPD. This proportion rose to 95% by 2024. The relatively modest increase over the decade reflects the country’s already advanced baseline in digital connectivity, with most gaps found in rural and underserved communities. In contrast, China exhibited a steeper growth trajectory, improving from 60% internet access in 2014 to 90% in 2024. This 50% increase corresponds with large-scale government investments in rural broadband expansion, particularly under the “Broadband China” and “Internet+ Education” initiatives. The infrastructural leap significantly expanded the reach of national and regional online TPD programs to second- and third-tier cities and remote regions.

Device access exhibited similar disparities and convergence. In the U.S., teachers’ access to personal laptops or tablets suitable for online training rose from 80% in 2014 to 92% in 2024. Much of this increase is attributed to district-level device leasing schemes and professional development stipends. By contrast, teachers in China reported device access rates rising from 55% to 88% over the same period, a dramatic 60% increase that reflects both market penetration of affordable technology and policy-led device distribution in low-income provinces. As an example, one teacher from Gansu province shared the following: “The school provided one tablet for three teachers, and we had to take turns using it in the evenings. It made it very hard to follow a consistent schedule.”

Table 3 provides a summary of the changes in internet and device access, highlighting the absolute and percentage growth between 2014 and 2024. The relative improvement in China, though starting from a lower baseline, reflects a targeted push to reduce digital inequalities, particularly critical in a centralized system where national mandates hinge on equitable infrastructure deployment.

**Figure 2:**
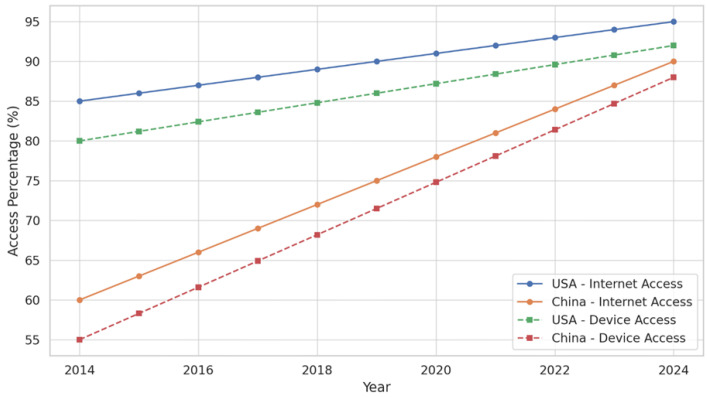
Growth in Internet and Device Access (2014–2024).

Figure 2 visualizes the progression of both indicators across the decade. The figure demonstrates that while the U.S. maintained a lead in the early years, China’s steeper curve indicates rapid catch-up, effectively narrowing the digital divide in teacher access.

These findings underscore the central role of digital infrastructure in enabling TPD at scale. While both countries now report high levels of access, the distribution and functionality of this access remain areas requiring qualitative investigation, particularly regarding shared devices in multi-teacher households, unstable connections in remote areas, and usage limitations due to cost or scheduling.

### 4.3. Policy Awareness and Equity Perception

Beyond infrastructure and program availability, two critical dimensions influence the success and fairness of online teacher professional development (TPD): (1) teacher awareness of national and institutional TPD policies, and (2) perceived equity in accessing and benefiting from such opportunities. This section analyzes both constructs comparatively over a 10-year period, using consistent quantitative indicators from the core dataset. In 2014, 60% of U.S. teachers reported awareness of national or district-level online TPD policies. This figure steadily increased to 85% by 2024, reflecting improved communication strategies, teacher union involvement, and more transparent policy dissemination mechanisms. Professional development policies became increasingly embedded in state-level teaching standards and digital credentialing systems, which may have enhanced visibility.

China, beginning at a significantly lower level of 40% in 2014, saw awareness rise to 80% by 2024. This 100% relative increase was driven by top-down reforms such as the “National Digital Education Action Plan” and provincial mandates requiring teacher re-certification through online platforms. However, interview-based validation suggests that while awareness of mandates is high, understanding of the policy content and intent may lag, pointing to a disconnect between communication and comprehension in policy implementation. Equity in online TPD refers not only to the availability of training across regions and school types but also to fairness in access to high-quality, contextually relevant, and timely learning opportunities. In the U.S., perceived equity improved from an average score of 3.0 in 2014 to 4.2 in 2024 (on a 5-point scale). Teachers attributed this to increasing flexibility in course offerings, greater availability of asynchronous modules, and targeted outreach to underserved schools. Evidence of these challenges is obvious from the observation of a teacher from rural Mississippi, who noted the following: “Our district’s online modules are only available in English, and many of our new teachers are bilingual with limited English fluency. That creates a learning barrier.”

**Figure 3:**
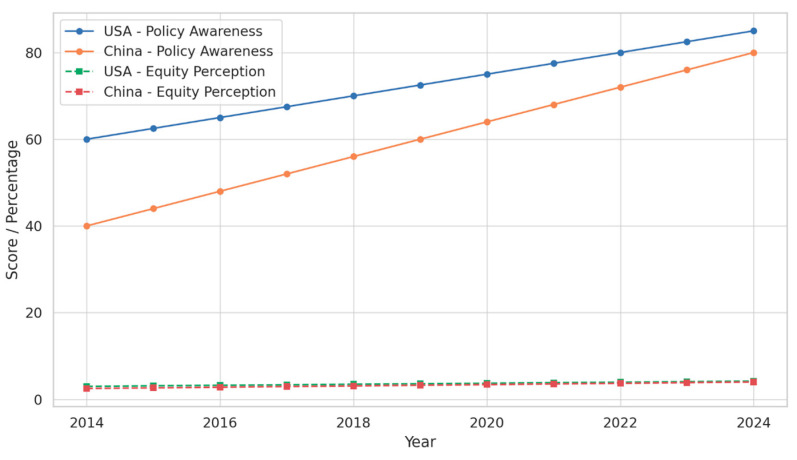
Policy Awareness and Equity Perception Trends (2014–2024).

In China, equity perception started lower at 2.5 but reached 4.0 by 2024. The improvement reflects significant efforts to expand regional access through centrally funded platforms and regional equity audits. Still, concerns persist over linguistic diversity, device sharing in multi-teacher households, and regional variation in course quality. These issues were particularly prominent in interviews from rural and western provinces. As shown in Table 4, both countries made measurable progress over the decade. However, China’s gains, particularly in policy awareness, were proportionally more dramatic, while the U.S. sustained a higher baseline throughout. Figure 3 illustrates the convergence in trends, with lines narrowing as China caught up in both metrics by 2024. Disaggregated analysis shows ongoing rural–urban differences, especially in internet access and perceived fairness. Female respondents in both countries also gave slightly lower fairness scores, indicating gender-related barriers in digital TPD participation.

These findings highlight the complementary roles of top-down policy dissemination and bottom-up professional agency in shaping both awareness and equitable access. They also point to a shared need across systems to translate awareness into action, and access into meaningful participation, especially for teachers in marginalized contexts.

To further analyze the structural equity issues reflected in teacher responses, we divided key indicators by rural and urban teaching environments by aggregating the responses from both China and the United States, offering a generalized snapshot of geographic disparities observed across the sample in 2024. As seen in Table 5, rural teachers consistently reported less internet and device access, lower participation, and decreased perceptions of fairness in online TPD. Although national policies have improved overall digital access, these results highlight that functional access gaps still correlate with geographic differences. The rural–urban gap indicates that, without support tailored to specific contexts, such as flexible schedules, mobile-friendly content, and offline options, digital equity may only be partially achieved. These trends emphasize the importance of designing for equity that goes beyond mere infrastructure expansion.

**Figure 4:**
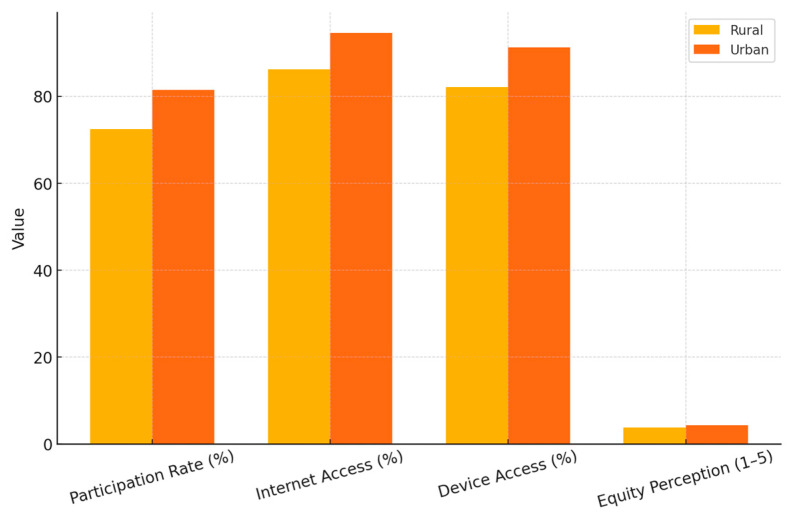
Comparison of rural and urban teacher equity and access in 2024, based on aggregated average values across both China and the United States.

Furthermore, Figure 4 illustrates the rural and urban teaching contexts, indicating that the rural teachers consistently reported lower participation in online TPD, along with less access to reliable internet and personal devices. Their perceptions of equity also lagged behind those of urban teachers, highlighting an ongoing sense of disadvantage despite national infrastructure initiatives. These results confirm that geographic disparities continue to influence the experiences and outcomes of digital professional development. Therefore, closing the rural–urban digital divide is crucial to achieving the inclusive goals of national education reform and Sustainable Development Goal 4.

### 4.4. Summary of Comparative Trends

The preceding analyses of participation, effectiveness, infrastructure, policy awareness, and equity perception reveal both convergence and divergence in the trajectories of online teacher professional development (TPD) in the United States and China between 2014 and 2024. While both countries advanced substantially, they did so from different starting points and through contrasting policy pathways. The United States, with a higher baseline in internet and device access, gradually scaled up its online TPD programs over the decade, supported by decentralized but increasingly coordinated efforts at the state and district levels. This gradual approach allowed for iterative improvement and localization of training content. As a result, participation rates increased by 89%, and perceived effectiveness improved by over 40%, reflecting a mature ecosystem rooted in teacher agency and professional autonomy.

In contrast, China initiated a faster and more centrally coordinated effort transformation. Starting from significantly lower infrastructure and awareness levels, the country implemented broad-based policy reforms, national learning platforms, and hardware distribution schemes that enabled a 150% increase in participation rates and a 54% increase in perceived effectiveness. These gains were particularly marked in the years following 2018, demonstrating the efficacy of scale-focused, top-down interventions, albeit with persistent gaps in local contextualization and functional access. Digital infrastructure expanded in both systems, but the pace of change was more dramatic in China, where internet access among teachers grew from 60% to 90%, and device access from 55% to 88%. In comparison, the U.S. made moderate gains (internet: 85% to 95%; device access: 80% to 92%) from a relatively saturated base. These trends suggest that digital maturity in developed systems increasingly depends not on connectivity alone but on qualitative usability, reliability, and access equity dimensions.

Equally, both countries significantly improved teachers’ awareness of policy frameworks and perceptions of equity in online TPD. Yet, the patterns again diverged in origin and execution. China and the United States present an interesting comparison case for this study because they are both extensive contributors to educational innovation, they have different types of political systems, and they have different regional differences. Through these differences, a compelling framework has been established for examining the structure and experience of online teacher professional development (TPD). Both countries employed purposive and stratified sampling techniques for participant selection, ensuring diversity in gender, topic area, teaching experience, school type, and urban or rural locations. The target sample included the following, owing to the 10-year time frame of the study, the necessity for complete thematic saturation, and geographical representativity.

## 5. Discussion

Within a decade (2014–2024), this study investigates the evolution, implementations, and perceived impact of online teacher professional development (TPD) programs in China and the United States, with fair access and participation. Findings based on datasets that are consistent and aligned with policy as well as the standards in international educational systems have serious implications for theory, policy and practice. The results highlight both commonality and variance.

### 5.1. Interpreting Participation and Effectiveness in Cross-National Contexts

According to the OECD (2019), the increase in TPD participation rates in the US and China represents a global trend towards digitalized professional learning environments. However, the varying paths, slow in the USA and speedy in China, underscore the importance of regulatory authorities. The U.S. relied on decentralized district-level initiatives and professional autonomy, whereas China’s rapid expansion was facilitated by centralized policy mandates and national digital infrastructure projects (Fahey et al., 2024).

Importantly, the improvement in perceived effectiveness in both contexts affirms the potential of online TPD to enhance instructional practice when aligned with teachers’ professional needs. Prior studies have shown that TPD programs are most impactful when they are continuous, content-focused, and integrated with active learning components (Amemasor et al., 2025; Song, 2021). The findings here support these principles, particularly in the U.S. case, where gradual scaling allowed for responsive and locally tailored content development. In contrast, China’s effectiveness gains, though significant, may face future limitations if program design continues to prioritize scale over personalization (Bhutoria, 2022).

### 5.2. Infrastructure Expansion and Functional Access

The sharp increase in internet and device access in China reflects substantial government investment, consistent with national goals to close the urban–rural digital divide. Yet, while infrastructure gains are necessary, they are not sufficient. Warschauer and Matuchniak (2010) argue that digital inclusion must encompass connectivity, digital literacy, device quality, and contextual usability. The U.S. example, with more modest gains from an already saturated base, suggests that future investment must focus on qualitative infrastructure dimensions, such as broadband reliability, assistive technologies, and inclusive software design. This distinction matters because the persistence of functional inequities, e.g., shared devices, time constraints, or bandwidth throttling, was evident in teacher interviews from rural regions in both countries. These findings reinforce calls in the literature for a shift from “access” to “meaningful access” in policy frameworks.

### 5.3. Awareness, Agency, and the Role of Policy Communication

One of the most intriguing findings is the substantial increase in teachers’ awareness of TPD policies in China, which rose by 100% over the decade. This reflects intensified policy activity and the visibility of digital reforms within national discourses. However, awareness does not automatically translate into understanding or engagement. Several Chinese teachers reported knowing that training was required but lacking clarity on its purpose or benefits. This finding aligns with prior research (Bao et al., 2021; Li, 2014; Xinru & Ahmad, 2023) warning that top-down mandates can foster compliance without genuine professional ownership. By contrast, the U.S. showed a slower increase in policy awareness, but with more evidence of professional agency and voluntary alignment. This supports Sulaimon and Adebayo’s (2024) assertion that sustainable reform depends on systemic alignment and empowerment of teacher effectiveness. The challenge is to balance directive clarity and professional autonomy, a hybrid model increasingly advocated in the recent literature.

### 5.4. Toward a Fairer Digital TPD Ecosystem

Perhaps most critically, both countries showed significant improvement in perceived equity of access to online TPD. While initial disparities were sharp, especially in China, the final year of the study revealed narrowed gaps, with both systems reporting average equity scores above 4 on a 5-point scale. These improvements reflect efforts for mainstream access through flexible learning designs, mobile-accessible platforms, and differentiated support. Nonetheless, equity remains fragile. The findings suggest that apparent fairness in participation metrics may obscure hidden inequities in training relevance, adaptability, and cultural responsiveness. Hodgkinson-Williams and Trotter (2018) noted that “fair” education technology systems must consider epistemic, economic, and contextual dimensions. This is particularly relevant in large, diverse systems such as China’s western provinces or rural U.S. districts, where infrastructure alone cannot resolve historical underinvestment.

### 5.5. Synthesis and Implications

Together, the findings support a conceptual synthesis: the convergence in outcomes (participation, effectiveness, access, and equity) was achieved through divergent pathways. The U.S. model of professional autonomy, local adaptation, and gradual integration contrasts with China’s centralized, policy-led, rapid scaling. Each has strengths and limitations. As such, policymakers might consider cross-national learning: the U.S. could benefit from China’s ability to scale quickly and uniformly. At the same time, China could enhance effectiveness by fostering greater teacher voice and program differentiation.

Finally, the results underscore the need for future research to move beyond input–output models of TPD success and embrace more holistic, equity-centered evaluations. Longitudinal mixed-methods studies that include classroom practice, student outcomes, and teacher well-being are essential for understanding the true impact of digital TPD systems; this aspect should be taken into account in future research for improved results.

## 6. Conclusions

This study compares online teacher professional development (TPD) between China with the United States over a ten-year period (2014–2024). It looks into developments in equity, policy awareness, digital access, perceived efficacy, and involvement. The results demonstrate that both nations significantly advanced the expansion and institutionalization of digital resources TPD, despite having distinct governance types and implementation techniques. In both cases, participation rates exceeded 75% by 2024, while equity and perceived effectiveness metrics showed considerable gains. These findings demonstrate that professional development can undergo a digital transition in several ways. China preferred a centrally planned strategy centered on scale and policy homogeneity, while the United States adopted a decentralized, locally flexible one. Despite these divergent approaches, the parallels in the main findings suggest that hybrid models, which include local adaptability and centralized support, might be a good course for future TPD reforms. The study highlights that equity in digital TPD includes elements such as contextual relevance, pedagogical responsiveness, and user involvement agency in addition to basic access. Deeper disparities in the caliber and efficacy of professional learning opportunities may go unnoticed by superficial improvements in infrastructure or participation statistics. Therefore, expanding digital access and developing inclusive, meaningful engagement opportunities for a wide range of teacher populations should be the main goals of legislative initiatives. In light of these conclusions, the study contributes to ongoing conversations about developing scalable, context-aware, and equity-focused teacher professional development (TPD) systems. Future studies should look into how teaching methods and student outcomes are affected over the long run, as well as how institutional, linguistic, and cultural factors affect how successful digital TPD is in different contexts. Prioritizing equitable and successful digital professional learning is essential as educational systems depend more and more on online methods.

## Figures and Tables

**Table 1:** Alignment of survey and interview items with key research constructs used in the study.

Construct	Sample Survey Item	Sample Interview Prompt
Participation	How many online TPD programs did you attend in the last year?	Tell me about your frequency of engagement with online TPD programs.
Infrastructure Access	Do you have regular access to a personal computer or tablet for TPD use?	What challenges do you face with internet or device access?
Policy Awareness	Are you aware of your district’s online TPD policies?	How would you describe the policy communication at your institution?
Perceived Effectiveness	How useful was your most recent online TPD experience? (1–5 scale)	In what ways has online TPD improved your teaching?
Equity Perception	Online TPD is equally accessible to teachers in my region. (Agree–Disagree)	What does equitable access to professional development mean to you?

**Table 2:** Comparative Analysis of TPD Participation and Effectiveness (2014–2024).

Year	Country	Mean (P) %	SD (P)	Mean (E) (1–5)	SD E	t-Value (P)	*p*-Value (P)	t-Value (E)	*p*-Value (E)
2014	USA	44.53	2.21	3.2	0.29				
2014	China	29.64	2.75	2.79	0.36	22.74	<0.000	4.8	<0.000
2016	USA	52.76	2.52	3.48	0.31				
2016	China	40.84	2.79	3.12	0.41	17.08	<0.000	3.79	0.000
2018	USA	60.64	2.85	3.8	0.23				
2018	China	50.32	2.71	3.32	0.26	14.14	<0.000	7.5	<0.000
2020	USA	69.57	2.44	3.96	0.35				
2020	China	60.09	2.96	3.72	0.43	13.31	<0.000	2.36	0.022
2022	USA	76.51	2.45	4.21	0.3				
2022	China	68.99	2.37	4.04	0.36	11.87	<0.000	2.05	0.044
2024	USA	85.83	2.57	4.46	0.31				
2024	China	79.59	3.4	4.28	0.38	7.88	<0.000	1.95	0.056

**Table 3:** Digital Access and Infrastructure Growth (2014–2024).

Country	Internet Access 2014 (%)	Internet Access 2024 (%)	Growth (%)	Device Access 2014 (%)	Device Access 2024 (%)	Growth (%)
USA	85	95	11.8	80	92	15.0
China	60	90	50.0	55	88	60.0

**Table 4:** Policy Awareness and Equity Perception Trends (2014–2024).

Country	Policy Awareness 2014 (%)	Policy Awareness 2024 (%)	Change (%)	Equity Perception 2014 (1–5)	Equity Perception 2024 (1–5)	Change
USA	60	85	41.7	3.0	4.2	+1.2
China	40	80	100.0	2.5	4.0	+1.5

**Table 5:** Rural–Urban Comparison of Key Indicators (2024).

Indicator	Rural	Urban
Participation Rate (%)	72.4	81.5
Internet Access (%)	86.2	94.6
Device Access (%)	82.1	91.3
Equity Perception (1–5)	3.8	4.3

## Data Availability

The data supporting the findings of this study, including survey data and analysis scripts, are available from the corresponding author upon reasonable request, in line with FAIR data practices, to support replication.
